# Reduced model to predict thrombin and fibrin during thrombosis on collagen/tissue factor under venous flow: Roles of γ’-fibrin and factor XIa

**DOI:** 10.1371/journal.pcbi.1007266

**Published:** 2019-08-05

**Authors:** Jason Chen, Scott L. Diamond

**Affiliations:** Department of Chemical and Biomolecular Engineering, Institute for Medicine and Engineering, University of Pennsylvania, Philadelphia, Pennsylvania, United States of America; University of Virginia, UNITED STATES

## Abstract

During thrombosis, thrombin generates fibrin, however fibrin reversibly binds thrombin with low affinity E-domain sites (K_D_ = 2.8 μM) and high affinity γ’-fibrin sites (K_D_ = 0.1 μM). For blood clotting on collagen/tissue factor (1 TF-molecule/μm^2^) at 200 s^-1^ wall shear rate, high μM-levels of intraclot thrombin suggest robust prothrombin penetration into clots. Setting intraclot zymogen concentrations to plasma levels (and neglecting cofactor rate limitations) allowed the linearization of 7 Michaelis-Menton reactions between 6 species to simulate intraclot generation of: Factors FXa (via TF/VIIa or FIXa), FIXa (via TF/FVIIa or FXIa), thrombin, fibrin, and FXIa. This reduced model [7 rates, 2 K_D_’s, enzyme half-lives~1 min] predicted the measured clot elution rate of thrombin-antithrombin (TAT) and fragment F1.2 in the presence and absence of the fibrin inhibitor Gly-Pro-Arg-Pro. To predict intraclot fibrin reaching 30 mg/mL by 15 min, the model required fibrinogen penetration into the clot to be strongly diffusion-limited (actual rate/ideal rate = 0.05). The model required free thrombin in the clot (~100 nM) to have an elution half-life of ~2 sec, consistent with measured albumin elution, with most thrombin (>99%) being fibrin-bound. Thrombin-feedback activation of FXIa became prominent and reached 5 pM FXIa at >500 sec in the simulation, consistent with anti-FXIa experiments. In predicting intrathrombus thrombin and fibrin during 15-min microfluidic experiments, the model revealed “cascade amplification” from 30 pM levels of intrinsic tenase to 15 nM prothrombinase to 15 μM thrombin to 90 μM fibrin. Especially useful for multiscale simulation, this reduced model predicts thrombin and fibrin co-regulation during thrombosis under flow.

## Author summary

During blood clotting events, a complex series of reaction are involved. Simulation gives insights to the concentration of different enzymes which are at too low of concentration to be detected. However, the models are often large and difficult to solve for clotting under flow conditions. With a thin film approximation, we were able to simplify clotting under flow with parameters from literature, with only 3 adjusted in order to fit the experimental data. This model gave insights into the dynamics of the species involved, and the roles of γ’-fibrin and thrombin feedback activation. This reduced model may be useful in further multiscale simulations.

## Introduction

The reaction network and kinetics of human blood clotting impact diseases such as coronary thrombosis, stroke, deep vein thrombosis, hemophilia, disseminated intravascular coagulopathy (DIC), and traumatic bleeding. Numerous therapeutics are designed to either inhibit or catalyze reactions of the coagulation cascade. Despite decades of study, new reaction modulators (eg. platelet polyphoshate [1] and reaction pathways (eg. direct conversion of FVIIIa by TF/FVIIa/Xa [2]) are still being discovered. In some cases, the significance of a particular reaction studied in a purified system may be difficult to resolve since pM-levels of factors formed transiently in whole blood are challenging to measure directly.

Excluding platelet metabolism other than the availability of anionic phospholipid, isotropic kinetic models of plasma coagulation in a closed system can include 50 to 100 reactions, 1 to 3 kinetic rate coefficients per reaction, and about 10 initial conditions for zymogen or cofactor concentrations [3–5]. Fortunately, these large ODE models can be parameterized and solved with minor computational expense. In these models, a trigger at t = 0 is required such as 1 to 10 pM tissue factor (TF) along with 1% of FVII being in a cleaved yet zymogen-like state as free FVIIa. Alternatively, if no TF is present, a source term for FXIIa generation or non-zero levels of cleaved factors is required to drive clotting [5]. In closed systems, the concentration of substrates and products can undergo >10^3^-fold changes as clotting proceeds non-linearly through initiation, propagation/amplification, and exhaustion (inhibition and substrate consumption). Calibrated automated thrombinography (CAT) assay reports these dynamics for platelet-poor or platelet-rich plasma with typical time lags of 3.1 and 8.1 min, peak thrombin levels of 458 and 118 nM at 10 min, and reaction completion by 25 min [6].

As clotting progresses, the extrinsic tenase/IXase (TF/FVIIa) converts FX to FXa and FIX to FIXa. Along with conversion of activated cofactors FVIIIa and FVa, the intrinsic tenase (FIXa/FVIIIa) dramatically amplifies production of FXa, while prothrombinase (FXa/FVa) generates thrombin (releasing fragment F1.2). Thrombin cleaves platelet PAR-1 and PAR-4 and converts fibrinogen to fibrin monomer by release of fibrinopeptides A and B (FPA/B). The reaction of thrombin and antithrombin to form thrombin-antithrombin (TAT) is relatively slow (~1 min) unless catalyzed by heparin. Fibrin monomers associate into protofibrils that laterally aggregate into bundles. Thrombin also activates FXIIIa, a transglutaminase that crosslinks fibrin. Plasmin-mediated fibrinolysis of crosslinked fibrin releases various fibrin degradation products (FDP) including D-dimer. These reactions can be studied in closed systems, ± fluid mixing and ± spatial gradients. To mimic thrombosis at a specific wall location (an open system), blood treated with the FXIIa inhibitor corn trypsin inhibitor (CTI) can be perfused over a defined thrombotic surface containing TF. Clotting on a surface under flow includes mathematically complex physical phenomenon such as platelet margination to the wall [7], convective/diffusive transport, concentration boundary layers, pressure-driven permeation, and moving boundaries [8,9]. Solving large sets of partial differential equations (PDEs) for coagulation species transport and reaction is expensive and non-trivial [10]. Generally, enzyme-substrate interactions at the single molecule level are considered unaffected by macroscopic flow forces.

Fibrin has ‘antithrombin-I activity’ via thrombin binding to the low affinity site in the E domain and the high affinity site in the D-domain of the alternative splice variant, γ’-fibrin(ogen). The γ’-fibrinogen splice variant represents about 6–8% of total γ-chains, with γA/γ’ heterodimer representing 12–16% of total fibrinogen [11]. γ′ fibrinogen level is associated with cardiovascular disease. [12]. During thrombosis under flow, thrombin co-localizes on fibrin [13,14]. A recent observation is that little thrombin (detected as TAT) leaks out of a growing clot unless fibrin polymerization is inhibited with Gly-Pro-Arg-Pro (GPRP) [15]. By immunoassays for TAT and F1.2 (± fibrin inhibitor, GPRP) and D-dimer (post-plasmin treatment), the dynamics of thrombin and fibrin generation have only recently been measured for flow of human whole blood over defined collagen/TF surfaces [13].

To our knowledge, no model has calculated intrathrombus thrombin generation and fibrin polymerization under flow conditions where fibrin is being formed dynamically and local thrombin is reversibly binding fibrin through the weak (E-domain) and strong binding sites (γ’-variant). We present a reduced model where key assumptions are supported by direct experiment measurements. This reduced model deploys a thin film assumption for the clot core (thickness ~ 15 microns) where zymogen levels in the clot are set to be identical to those in the flowing plasma. This assumption did not hold for fibrinogen transport, which is not surprising given that fibrinogen (340 kDa) is considerably larger than the other coagulation factors. For a set of prevailing plasma concentrations for Factors FVIIa, FIX, FX, FXI, prothrombin, fibrinogen as well as initial surface [TF]_o_, the reduced model makes quantitatively accurate predictions of thrombin and fibrin levels under venous flow conditions. Fibrin appears to allow for explosive but feedback-inhibited production of thrombin. After a clotting episode, the large amount of fibrin-bound thrombin was predicted to take a few hours to elute into the circulation to form TAT. This reduced model may be particularly useful for multiscale simulations of thrombosis over vessel length scales of mm to cm.

## Results

### Thrombin and fibrin production for blood flow over collagen/TF

**Fig 1:**
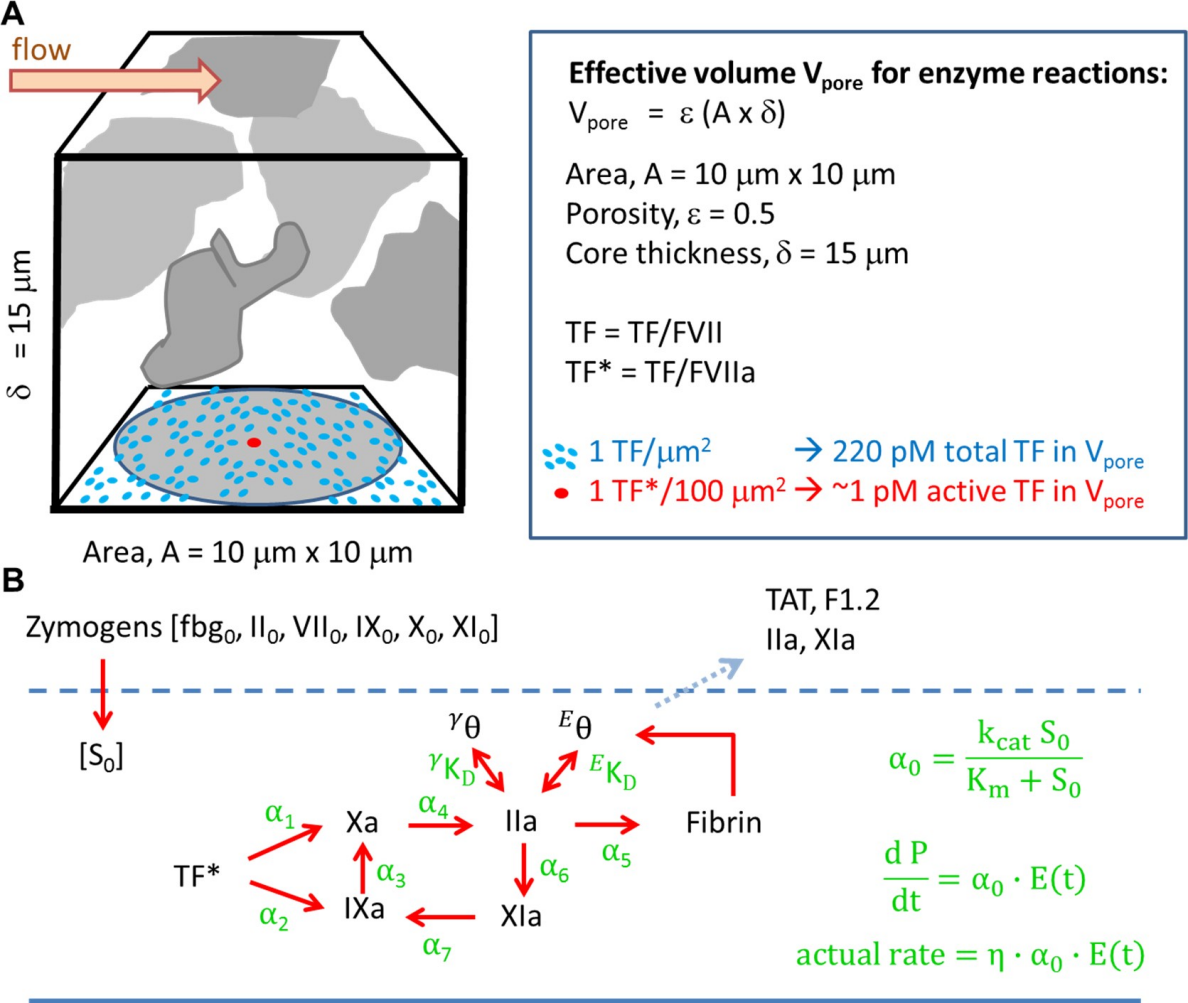
Schematic of the simplified ODEs model. The concentration of active TF* is defined as TF/FVIIa which is homogenized over the porous core volume V_pore_ (A). All zymogens were assumed to enter the clot core by diffusion to maintain their plasma level [S]_o_. All active enzymes had a 1-minute half-life, with TF* set to 3 min (since FVIIa generation was ignored). Free thrombin and FXIa eluted by diffusion from the core with a 2-sec half-life. The thrombin core thickness was set to 15-μm, with 50% of platelets by vol. Only the activated proteases are shown for simplicity.(B).

**Fig 2:**
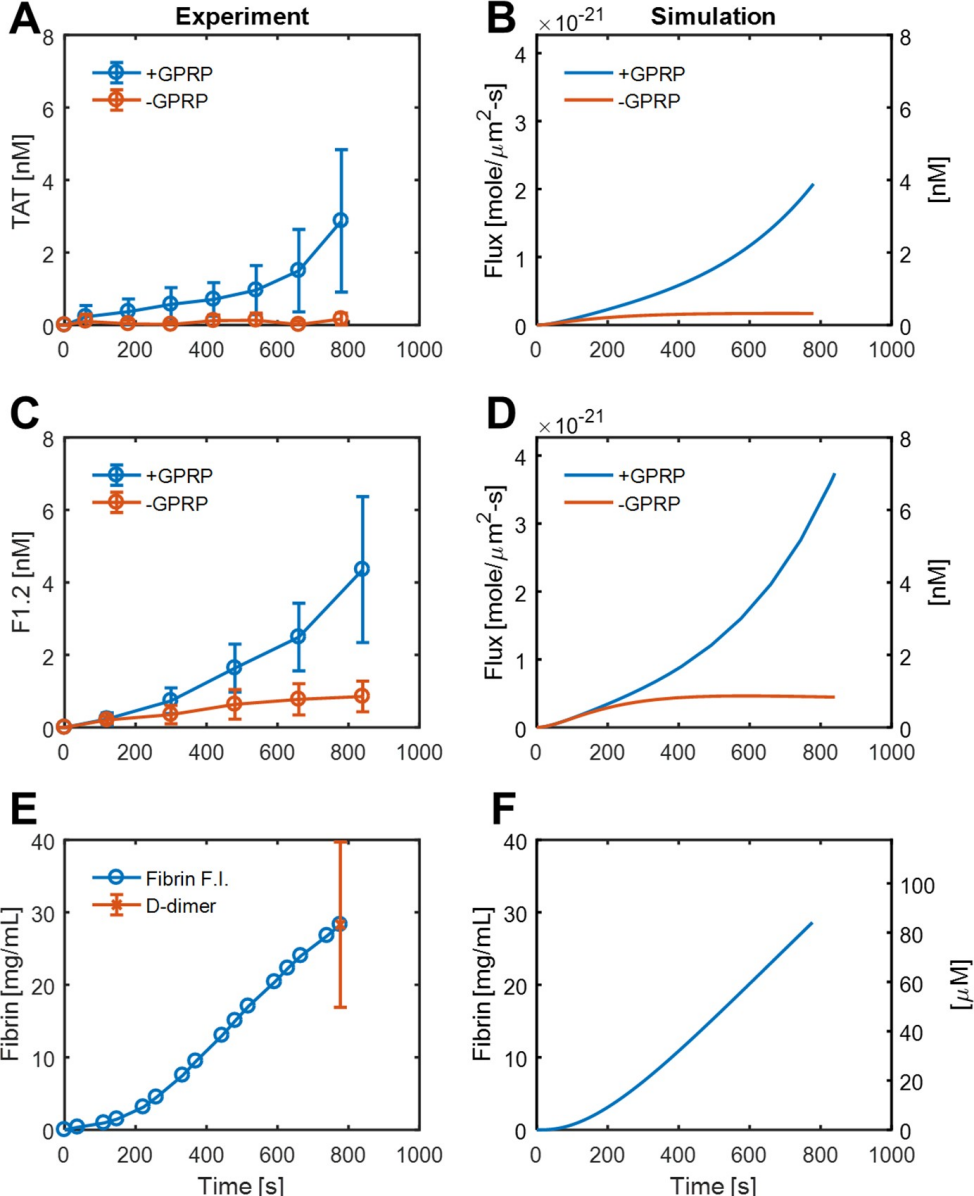
Comparison of experiment and simulation for TAT, F1.2, and fibrin dynamics. Thrombin-antithrombin (TAT) and Fragment F1.2 elution from clots in the presence or absence of fibrin (± GPRP) for experimental perfusion of whole blood over collagen/TF (A,C) and in simulations under identical conditions (B, D). Fibrin was measured dynamically by fluorescent fibrinogen incorporation and then calibrated by end-point D-dimer assay following plasmin degradation (E), while the intrathrombus fibrin concentration was simulated (F).

For perfusion of CTI-treated whole blood across a 250-μm long patch of collagen/TF (1 molecule-TF/μm^2^), platelets rapidly accumulate and create a sheltered reaction environment triggered by TF for production of thrombin and fibrin [15,16]. The effluent can be sampled and subjected to immunoassays to determine the measured species flux for a 250-long x 250 μm-wide patch of collagen/TF for TAT and F1.2, in the presence and absence of fibrin assembly (± GPRP) (**Fig 2A** and **2C**). The dynamic accumulation of fluorescent fibrin in the experiment was converted to a fibrin concentration by end-point immunoassay of D-dimer, post-plasmin treatment (**Fig 2E**). For the 7 reaction rate coefficients (α_1_-α_7_) (**Fig 1B, Table 1**), only 3 rates required adjustment (η_4_, η_5_, η_6_) from their literature values in order to simulate thrombin and F1.2 elution and fibrin polymerization in the presence and absence of GPRP. The adjustments for prothrombinase activity (η_4_ = 0.18) and thrombin activation of FXIa were modest (η_6_ = 0.36) and could involve either transport rate limits or just as possible the difference of the reaction in the whole blood milieu in comparison to dilute buffer conditions used in enzyme studies. The adjustment in thrombin mediated activation of fibrinogen was markedly pronounced, requiring a 20-fold reduction in the rate (η_5_ = 0.05). This 20-fold reduction in rate corresponds either to a ~80-fold increase in K_m_ (unlikely) or an 80-fold decrease in the intraclot level of fibrinogen substrate relative to plasma levels. In the experimental measurement, the generation of fibrin per thrombin molecule was unexpectedly low, given the known speed of FPA release by thrombin (k_cat_ = 80 s^-1^). In considering the value η_5_ as an effectiveness factor (actual rate/ideal rate in the absence of transport limits), the penetration of fibrinogen (340 kDa) into the dense fibrin-rich core of the clot is hypothesized, and required in the model to be diffusion-limited.

**Table 1:** Reactions and kinetic parameters used in the ODEs model.

#	Reactions	Enzyme	[S]_0_	k_cat_ (s^-1^)	K_m_ (μM)	α (s^-1^)	η	Ref.
1	$X\overset{{TF}^{*}}{\to }Xa$	TF/VIIa	X_0_ = 0.17 μM	1.15	0.24	0.48	1	[17]
2	$IX\overset{{TF}^{*}}{\to }IXa$	TF/VIIa	IX_0_ = 0.09 μM	1.8	0.42	0.32	1	[5]
3	$X\overset{IXa}{\to }Xa$	IXa/VIIIa	X_0_ = 0.17 μM	8.2	0.082	5.53	1	[5]
4	$II\overset{Xa}{\to }IIa$	Xa/Va	II_0_ = 1.4 μM	30	0.3	24.7	0.18	[5,17,18]
5	$\alpha -fbg\overset{IIa}{\to }desA-Fn1+FPA$	IIa	α-fbg_0_ = 18 μM	80	6.5	58.8	0.05	[5,19]
6	$XI\overset{IIa}{\to }XIa$	IIa/p*	XI_0_ = 31 nM	1.3x10^-4^	0.05	4.98x10^-5^	0.36	[5]
7	$IX\overset{XIa}{\to }IXa$	XIa/p*	IX_0_ = 0.09 μM	0.21	0.2	0.065	1	[5,20]
	thrombin binding to fibrin			K_d_ (μM)	k_f_ (μM^-1^s^-1^)	k_f_ (s^-1^)		
1	IIa + E site ↔ IIa ∙ E site			2.8	100	280		[21]
2	IIa + γ site ↔ IIa ∙ γ site			0.1	100	10		[21]

Simplified clotting reactions neglecting limits in activated cofactor generation, plasma zymogen concentrations, and kinetic parameters of coagulation where η is the effectiveness factor (actual rate with transport limits/theoretical maximum rate). For each reaction, α_o_ = k_cat_ [S]_o_/(K_m_+[S]_o_). Reversible binding of thrombin to the weak and strong site in fibrin was treated as kinetically-controlled, reversible adsorption.

The model clearly predicts that thrombin has difficulty eluting from the fibrin due to fibrin binding (**Fig 2A and 2B**). Once fibrin is prevented from forming or binding thrombin (α_5_ = 0 or setting K_D_>10 M) in the simulation or in the experiment (+GPRP), the TAT flux increases linearly with time for the first 500s and then increases even faster from 500 to 800s. As thrombin is generated, a small fragment F1.2 is released as a result of the prothrombinase activity. In both experiment and simulation, F1.2 elutes from the clot even in the presence of fibrin (**Fig 2C and 2D**). Additionally, more F1.2 is made than TAT, since thrombin can be inhibited by other inhibitors such as C1 and α_2_-macroglobulin; the simulation accounts for this (Note the value of 0.7 in Eq 1 for J-F1.2). With GPRP to eliminate fibrin’s antithrombin-I activity and facilitate FXIa-mediated feedback pathway, more F1.2 is detected both in the experiment and in the simulation (**Fig 2C and 2D**). Under flow conditions, fibrin reached a concentration that was 10-fold greater than plasma fibrinogen concentration (3 mg/mL, 9 μM) (**Fig 2E**).

### Dynamics of intrinsic tenase and prothrombinase

**Fig 3:**
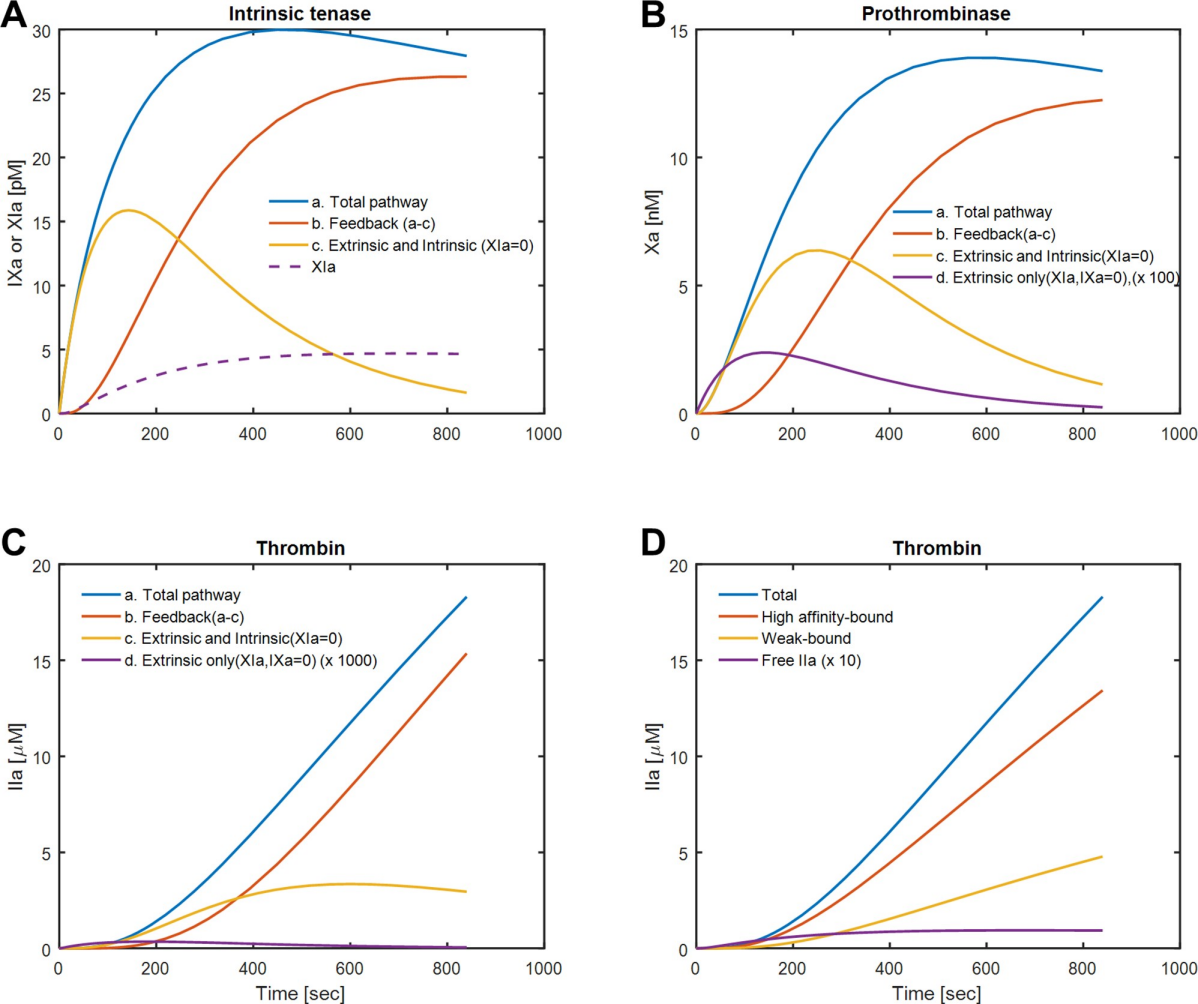
Concentration of the procoagulants predicted by the ODEs model. The concentration of FIXa (ie the intrinsic tenase, FIXa/FVIIIa) generated by all pathways (a,blue), in the absence of feedback with FXIa = 0 (c, orange) and by the FXIa-feedback pathway (b, red) calculated via a-c. (A). FXIa is shown as dashed-line. The concentration of FXa (ie. Prothrombinase, FXa/FVa) generated by various pathways (B) demonstrating that only a minor fraction of FXa is derived from TF/VIIa in the simulation. The concentration of thombin generated via various pathways (C). The majority of intrathrombin thrombin is bound to the γ’-site in fibrin with <100 nM as free thrombin (D).

The dynamics of intrinsic tenase generation, prothrombinase production, and thrombin binding to fibrin were explored in the model under various conditions. In the model, intrinsic tenase (“IXa” = FIXa/FVIIIa) reaches a level of 30 pM by 200 sec. By turning off thrombin-feedback activation of FXIa (setting α_6_ = α_7_ = 0), the model demonstrates that most of the intrinsic tenase is generated in the first 200 sec is from tissue factor (**curve *c*, Fig 3A**) while after 500 sec, most of the intrinsic tenase is a result of the feedback activation of FXIa by thrombin as seen in curve b = (a–c) (**Fig 3A**). FXIa reaches a level of only 5 pM in the simulation (**dashed line, Fig 3A**) demonstrating how potent FXIa can be for FIXa production and thrombin production. Similarly, the intrinsic tenase can be turned off (i.e. severe hemophilia) by setting α_2_ = α_3_ = 0 such that all of the prothrombinase is the direct result of the extrinsic tenase (**Fig 3B**). In this case, very little prothrombinase is generated, as expected for extreme hemophilia A/B. The role of FXIa in prothrombinase generation can be seen, especially after 500 sec, where most of the prothrombinase is a downstream result of the generation FXIa (**Fig 3B**). In the simulation, little thrombin is made when the extrinsic tenase/FIXase (TF*) cannot generate FIXa (α_2_ = 0), again consistent with the circumstances of severe hemophilia. As expected from the dynamics for prothrombinase, the majority of thrombin made at times >500 sec was the result of thrombin-feedback activation of FXIa (**Fig 3C**). Thrombin reached 18 μM-levels after 800 sec of clotting with almost all of it bound to the weak (E-domain) and strong (γ’) site and about only 1% of the thrombin (~100 nM) existing as a free species (**Fig 3D**). By 800 sec of clotting, the full effect of cascade amplification is seen in that an initial surface concentration of [TF]_o_ = 1 molecule-TF/μm^2^ (2.2 pM TF/VIIa = TF* in the core) results in the generation of 30 pM intrinsic tenase, ~15 μM prothrombinase, ~18 μM thrombin (100 nM free thrombin), and ~90 μM fibrin (30 mg/mL).

### Role of γ’ fibrinogen level

**Fig 4:**
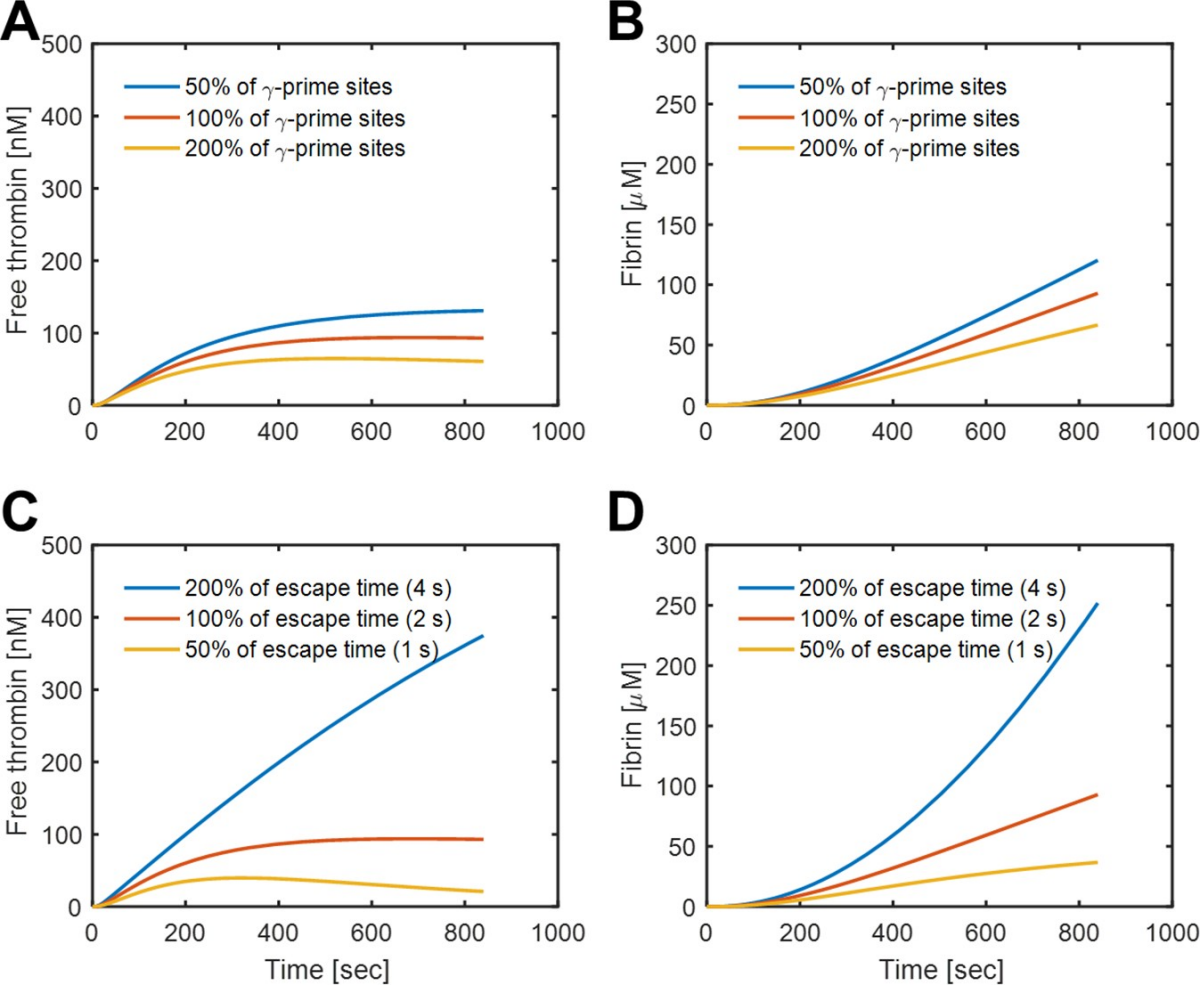
Effect of γ’ site concentration and escape time on thrombin and fibrin. The thrombin (A) and fibrin concentration (B) at 50%, 100%, and 200% of normal levels of **γ**’-fibrinogen. The thrombin (C) and fibrin concentration (D) for different diffusional escape times of free thrombin from the clot.

The range of **γ**’ fibrinogen concentrations can vary in healthy individuals [22], with a reference range of 0.088 to 0.551 mg/mL. Additionally, fibrinogen is an acute response gene and the fraction of splice variant can change. The concentration of **γ**’ fibrinogen concentrations and the **γ**’ fibrinogen/total fibrinogen ratio have been reported to be relevant in thrombosis, and different in different stages of disease, potentially with some protectant effect in venous thrombosis [23]. In the simulation, we varied the **γ**’ fibrinogen concentration to explore the effect on the co-regulation of fibrin and free thrombin concentration. With more **γ**’ fibrinogen, there was slightly more high-affinity sites for thrombin, therefore, sequestering more thrombin and decreasing the fibrin and free thrombin concentration (**Fig 4A and 4B**). In contrast, a 50% reduction in ^γ^θ caused a slight increase in the level of free thrombin and the amount of fibrin made. However, the effect of **γ**’-fibrinogen levels were not particularly marked, a reasonable result given the excess fibrin that is formed relative to thrombin, but still suggestive of a protective or regulating contribution in venous thrombosis.

### Protein escape time

As reported previously, platelet contraction can alter protein transport [24] with soluble proteins retained longer in the core of the clot than the less dense outshell shell. In the laser injury mouse model, albumin half-life in the clot core has been measured to be about 2 sec. In the simulation, we artificially adjusted the escape time between 1 sec and 4 sec to explore how intrathrombus diffusion influences local free thrombin and, consequently, fibrin production. A longer escape time of 4 sec resulted in dramatically higher intrathrombus concentration of fibrin and free thrombin, indicating the model was very sensitive on escape time. The concentration of thrombin increased more than 3-fold with a doubled escape time to 4 sec (**Fig 4C and 4D**).

### Convection-diffusion simulation of thrombin loading and elution from fibrin

**Fig 5:**
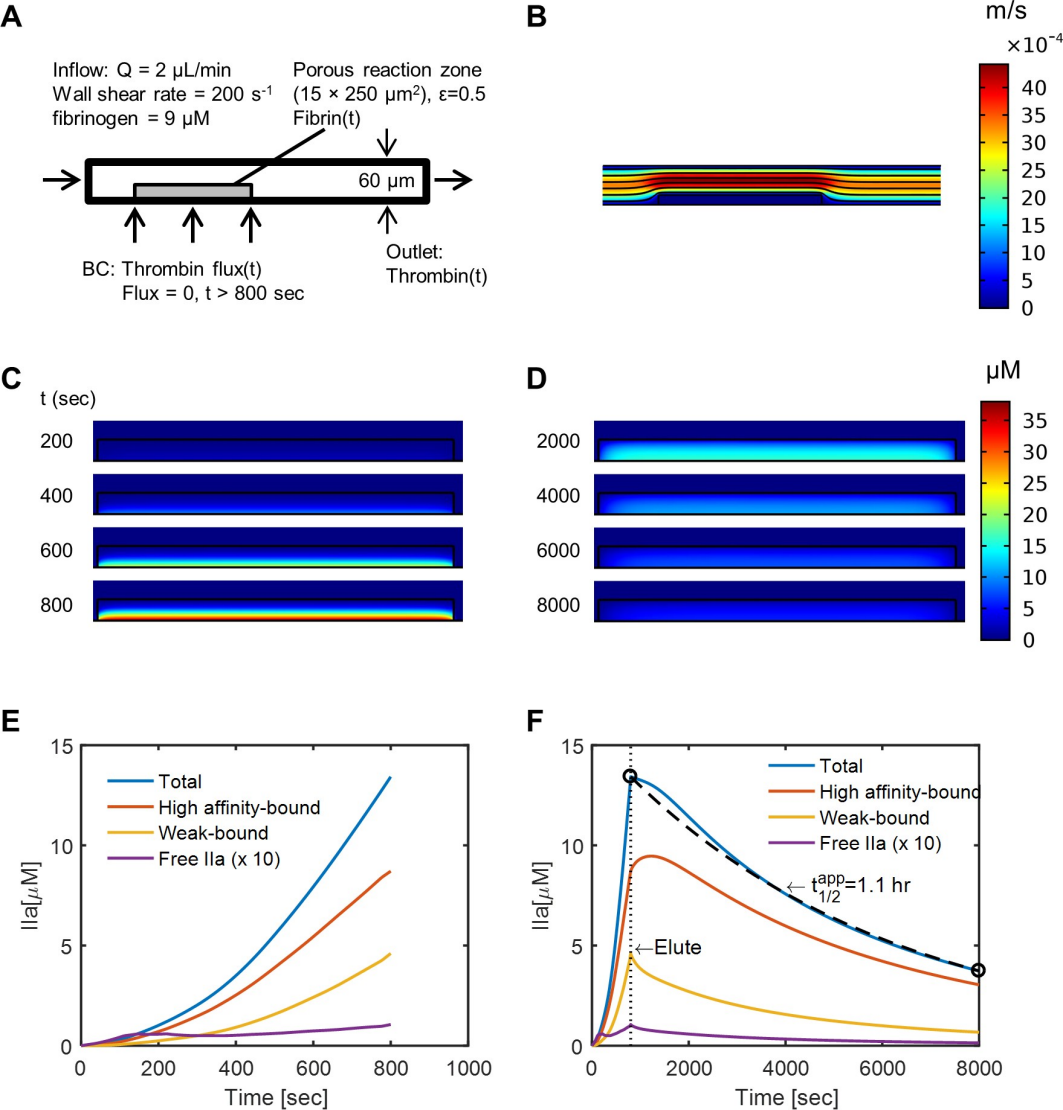
Transient convection-diffusion of thrombin into and out of a fibrin domain exposed to venous flow. The 2D simulation domain and imposed boundary conditions (A) allowed determination of the velocity field (B) and the intrathrombin thrombin transport dynamics over time (C, D). The average concentration of total, free, and bound thrombin in the clot domain are shown for imposed thrombin flux and fibrin concentrations (E). After 800 sec, no fresh thrombin was delivered into the clot and the elution of thrombin from the clot domain was followed (F).

To simulate dynamic concentrations of intrathrombus thrombin in the core, the velocity field and convective-diffusive transport of thrombin was calculated by COMSOL (**Fig 5A and 5B**). The empirically measured flux of thrombin J_IIa_(t)|_Y = 0_ (via F1.2 ELISA) was set at the bottom boundary condition of the core region. The empirically measured time-varying fibrin concentration fibrin(t) (calibrated by end-point D-dimer ELISA) was set uniformly in the core region. The concentration of ^E^θ and ^γ^θ sites were set to 1.6x fibrin(t) and 0.3x fibrin(t), respectively. After 800 sec, the thrombin flux entering the domain was set to zero in order to explore long term thrombin elution from the clot. The time-averaged flux into and out of the clot outlet (**Fig 5C and 5D**) revealed that most of the thrombin was captured by the fibrin, via both sites. By 500 sec, the concentration of intrathrombus thrombin was only 61 nM, only about 1% of total thrombin (5.5 μM) in the clot (**Fig 5C and 5D**), indicating that the literature K_D_ values for binding were consistent with actual independent measurements of TAT elution. The transient concentrations of total thrombin, intrathrombus free thrombin, and bound thrombin to each site, are shown in **Fig 5E**.

After 800 sec, the thrombin flux from the bottom plate was set to zero and the thrombin in the clot was allowed to be eluted by diffusion under prevailing flow conditions. The binding of thrombin by fibrin was sufficiently strong under a venous shear rate with an apparent half-life in the clot of 1.1 hour (**Fig 5F**). Thrombin eluted slowly into the flow field, relative to its half-life in the presence of antithrombin, such that its concentration would not be expected to perturb the hemostatic balance in the circulation. Thus, circulation levels of TAT can accumulate over hours and be measured in patients, even when most of the thrombin made in the first 800 sec is fibrin bound.

## Discussion

By assuming plasma zymogens can enter a thin clot at a rate significantly greater than their consumption, a highly reduced and essentially linearized ODE model provided a reaction topology suitable for accurate prediction of blood clotting on collagen/TF under venous flow. The thin film assumption was first formalized in Kuharsky-Fogelson model [25] to generate a large systems of ODEs describing clotting under flow. With the well mixed, thin film approximation, we were able to simplify clotting under flow to 8 ODEs and 19 parameters. A total of 16 parameters were from literature and only 3 were adjusted in order to fit the measured TAT and F1.2 and fibrin generation data (± GPRP). Of the 3 adjustable parameters, only the rate of fibrinogen activation by thrombin appeared to be strongly diffusion-limited (η_5_ = 0.05). This result was not particularly surprising given the enormous size of fibrinogen in comparison to the other coagulation factors. While ignoring cofactor activation of FVa and FVIIIa as non-rate limiting appeared to be compatible with predicting clotting of healthy blood, the generation of FIXa was absolutely required for robust thrombin production.

As an ODE model, the actual transport physics were mainly parameterized by the rate of free thrombin elution from the clot, guided by experimental measurements of ~2-sec half-life of flash-activated albumin in a clot subjected to flow along its outer boundary. In the presence of thrombin binding to fibrin, the elution rate of free thrombin from the clot appears to be an important regulator of clotting (**Fig 4C and 4D**). The 2-sec elution half-life for proteins was consistent with (i) in vivo mouse measurement, (ii) the human blood microfluidic measurements, and (iii) the average time it takes a protein to diffuse an average distance of 15 microns.

The roles of FXIIa in mouse thrombosis models [26] and platelet released polyphosphate to amplify thrombin-mediated feedback activation of FXIa [27] have motivated the pharmaceutical development of FXIIa, FXIa, and polyphosphate inhibitors. In **Fig 3A**, FXIa reaches ~5 pM by 500 sec of clotting and the amount of thrombin generated between ~500 and 800 sec is largely FXIa-dependent (**Fig 3C**). This is exactly consistent with microfluidic experiments conducted with anti-FXI antibody that blocked the increase of TAT and F1.2 flux and fibrin deposition after 500 sec [13,15,27].

By calibrating the model on measured thrombin and fibrin generation rates, the simulation provides insights, based on Table 1 kinetics, into pathways proximal to thrombin. The concentrations of FIXa/FVIIIa and FXa/FVa and FXIa were particularly low and would be difficult to measure directly inside the clot under flow conditions. Over 800 sec of clotting, the model revealed “cascade amplification” from 30 pM levels of intrinsic tenase to 15 nM prothrombinase to 15 μM thrombin to 90 μM fibrin, with FXIa pathways contributing significantly after 500 sec. Interestingly, little thrombin results directly from the FXa produced by TF/FVIIa, consistent with severe hemophilic blood producing little fibrin following perfusion over TF surfaces [28].

For the thin core region within the rapidly formed platelet deposit, the kinetics of thrombin and fibrin production are largely sheltered from the prevailing flow on the outer boundary of the clot. The current model may have some applicability to core dynamics during TF-driven arterial thrombosis since the core thickness (thrombin and fibrin and P-selectin positive region) has been measured to be relatively similar between the venous (100 s^-1^) and arterial (2000 s^-1^) condition [29].

As a model analyzing dynamics limited to the thin, core region using ODEs, the model was not designed to predict clot growth and spatial dynamics over distances of 100s or 1000s of microns. However, the TF-dominated thrombin generation rate in the core region could be coupled to spatial models of platelet deposition and FXIa-enhanced thrombin generation. The reduced model focuses on concentration changes of thrombin and fibrin in the thin “core” region which is only 15-microns thick. Since proteins can diffuse this short distance in ~10 sec, the well-mixed assumption of this thin region is reasonable, although not suited for predicting longer distance wave propagation such as found for clotting in stagnant plasma over millimeter distances [30–32].

The full biochemical and spatial complexity of human coagulation (typically involving >50 reactions in reality and in simulation) may relate more to the kinetic demands of hemostasis and its strong selective pressure (eg. surviving child birth), rather than to the complexities of thrombosis in older adults of modern times. The reduced model exploits the thin-film approximation under flow to emphasize a few key zymogen activation events at constant zymogen concentration. Despite its simplicity, this reduced model may have a useful implementation within more complex spatial thrombosis models that include platelet activation and accumulation [33,34].

The reduced model is not directed at describing the full progression of a thrombotic event over large spatial distances in large vessels, particularly where platelet accumulation dominates the growth process. However, this model may be very useful in establishing a surface TF-dependent thrombin flux and fibrin regulation that is a time-dependent boundary condition to a larger multi-species PDF model where platelets continue to accumulate and thrombin production transitions to platelet polyphosphate/FXIa-dependent.

Few simulations of clotting under flow include the role of anti-thrombin-I activity of fibrin or γ’-fibrinogen levels. The ratio of γ’-fibrinogen to total fibrinogen may be clinically relevant. Reduced γ’-fibrinogen levels have been associated with an increased venous thrombosis risk [23,35] Here, we demonstrated a simplified ODEs model to simulate the thrombin and fibrin generation. This reduced model may be particularly useful in multiscale simulations that seek to account for single platelet phenomenon [36], microscopic attributes of a wound site [16], and whole vessel dynamics [17,34].

## Methods

A reduced kinetic model of coagulation under flow was formulated to include extrinsic tenase/FIXase activity, intrinsic tenase activity, prothrombinase activity, feedback activation of FXIa by thrombin, fibrin generation, and thrombin binding to fibrin (**Fig 1**) using measured Michaelis-Menton kinetic parameters (Table 1). The reduced model employs various physical and biochemical features of clotting under flow that are supported by experimental measurement:

### 1) Core thickness

By direct imaging, blood clotting over collagen/tissue factor surfaces generates a 15-micron thick “core” region (δ = 15 μm, porosity ~0.5) that contains P-selectin-positive platelets with concomitant thrombin generation and dense fibrin deposit [13,37,38]. This is the thin film reaction compartment in which species are considered to be pseudo-homogeneous and of uniform concentration. The porosity used in the model is an estimate of the spatially averaged porosity over the entire 250 μm x 250 μm clotting region of the microfluidic assay, recognizing that this averages over both platelet/fibrin dense regions surrounded by fibrin dense regions. It is expected that the lower porosity decreases the effective diffusion of thrombin.

### 2) Substrate delivery

Substrate concentrations in the clot core are considered to be constant at plasma levels (S_o_). Thus, the Michaelis-Menton reactions to generate product [P] become linearized with respect to [E, enzyme] as dP/dt = [k_cat_ (S_o_/(K_m_+S_o_))] • E(t) = α• [E(t)] (**Table 1**). For species without diffusion limitations, the effectiveness factor ƞ = 1 (actual rate/ideal rate without diffusion limits). If a species experience transport limits, then ƞ < 1 and dP/dt = ƞ • α• [E(t)]. This approach is supported by direct measurements of TAT, F1.2, FPA, and D-dimer that indicate local clot associated product levels (thrombin and fibrin) are in excess of plasma levels (prothrombin and fibrinogen) demonstrating continual substrate delivery into the core of the clot [13].

### 3) Product escape from the clot core

Just as substrates can enter the core, free thrombin and FXIa were considered to escape the clot. The escape time was set to the measured half-life of 2 sec for albumin within the clot core [24] where k_elute_ = ln(2)/2 sec. This half-life in the thin-film for product escape = k_elute_ • [E] is conceptually and mathematically similar to the use of a mass transfer coefficient k_c_ with units of 1/time as defined in [25].

Although the binding characteristics of F1.2 to fibrin are unknown, we hypothesize that the observation that fibrin suppresses F1.2 elution may be consistent with fibrin inhibiting the thrombin-feedback pathway involving FXIa which in turn results in less prothrombin conversion. The small Fragment F1.2 was considered to leak out of the clot core as fast as thrombin was generated in the core. For TAT, 70% of the thrombin eluted from the clot was considered complexed with antithrombin with the remaining 30% of eluted thrombin complexed with other inhibitors [13]. Based upon all thrombin and F1.2 begin generated in the pore space V_pore_ of the clot core (**Fig 1A**), the flux J-F1.2 and the flux J-TAT leaving the clot were calculated as:

$$Flux,\;J‐F1.2\left(t\right)=\eta_{4}•\alpha_{4}\left[Xa\left(t\right)\right]\left(V_{pore}/area\right)=\eta_{4}•\alpha_{4}\left[Xa\left(t\right)\right]\delta$$ Eq 1

$$Flux,\;J‐TAT\left(t\right)=0.7•k_{elute}\left[IIa\left(t\right)\right]\left(V_{pore}/area\right)=0.7•k_{elute}\left[IIa\left(t\right)\right]\delta$$ Eq 2

### 4) Cofactors not rate limiting

For healthy non-hemophilic blood, the generation of cofactors (FVa, FVIIIa) was treated as non-rate limiting. Thus, the intrinsic tenase FIXa/FVIIIa = “FIXa”, and prothrombinase FXa/Va = “FXa”. The availability of FIXa and FXa (not FVa or FVIIIa) controlled the enzymatic cleavage of their substrates according to the reactions parameterized in Table 1.

### 5) Initial surface concentration

FVII and FVIIa in plasma were assumed to instantaneously equilibrate with surface TF such that [TF*] = TF/FVIIa = 1% of [TF]_0_ where [TF]_0_ = 1 molecule/μm^2^ set experimentally. No additional TF* was allowed to be generated in the model, equivalent to the quenching dynamics via platelet coverage invoked by Kuharsky and Fogelson. [25].

### 6) Order of 1-minute enzyme half-lives

The inhibition mechanisms of coagulation proteases via TFPI, ATIII, C1-inhibitor and α_2_-macroglobulin are complex and diverse and not fully resolved. Inhibition was treated uniformly to be a pseudo-first order reaction. Enzyme half-lives were set to be on the order of 1-min for FXa, FIXa, FXIa, FIIa (and 3 min for TF* since FVIIa generation was neglected). In other words, inhibition was clearly not as rapid as 0.1 min and clearly not as slow as 10 min. Thus, k_i_ = ln(2) /60s as a first approximation.

### 7) Thrombin adsorption to fibrin

Reversible thrombin binding to the weak E-domain site (^E^K_D_ = 2.8 μM) and the strong γ’-site (^γ^K_D_ = 0.1 μM) was consider to have diffusion-limited association (k_f_ = 100 μM^-1^ s^-1^). Fibrin-bound thrombin was considered to be fully resistant to inhibition. All fibrin monomer generated in the clot core was assumed to be fully incorporated into fibrin.

The delivery of plasma zymogens and platelets to the surface of a growing clot would be even faster at arterial flow conditions, however the increased shear forces tend to enhance platelet removal. Unfortunately, it is difficult to measure eluted FPA, F1.2, or TAT under arterial conditions due to their 10-20X greater dilution in the exit flow stream compared to the venous measurement [15]. Importantly, arterial syndromes tend to be drugged with anti-platelet agents, not anticoagulants.

For the reaction topology shown in Fig 1, these assumptions result in a reduced clotting model with only 8 ODEs for 6 reactive species undergoing 7 reactions (Table 1) and 2 fibrin sites for reversible binding of thrombin. These 8 ODEs were solved in Matlab R2016b using the ODE solver ode15s.

ODE 1. $\frac{d\;{TF}^{*}}{dt}=-k_{i,TF}∙{{TF}^{*}}^{}\;for\;k_{i,TF}=ln(2)/180s$

ODE 2. $\frac{d\;Xa}{dt}=\alpha_{1}∙{TF}^{*}+\alpha_{3}∙IXa-k_{i}∙Xa$

ODE 3. $\frac{d\;IXa}{dt}=\alpha_{2}∙{TF}^{*}+\alpha_{7}∙XIa-k_{i}∙IXa$

ODE 4. $\frac{d\;XIa}{dt}=\eta_{6}{∙\alpha }_{6}∙IIa-k_{elute}∙XIa-k_{i}∙XIa\;for\;\eta_{6}=0.36$

ODE 5. $\frac{d\;Fibrin}{dt}={\eta_{5}∙\alpha }_{5}∙IIa\;for\;\eta_{5}=0.05$

where: ${}^{E}\theta_{total}=(1.6)∙fibrin$ and ${}^{\gamma }\theta_{total}=(0.3)∙fibrin$

ODE 6. $\frac{d\;IIa}{dt}={\eta_{4}∙\alpha }_{4}∙Xa-(\frac{d^{E}S}{dt}+\frac{d^{\gamma }S}{dt})-k_{elute}∙IIa-k_{i}∙IIa\;for\;\eta_{4}=0.18$

ODE 7. $\frac{d^{E}S}{dt}={}^{E}k_{f}∙\mathrm{IIa}∙({}^{E}\theta_{total}-{}^{E}S)-{}^{E}k_{r}∙{}^{E}S$

ODE 8. $\frac{d^{\gamma }S}{dt}={}^{\gamma }k_{f}∙\mathrm{IIa}∙({}^{\gamma }\theta_{total}-{}^{\gamma }S)-{}^{\gamma }k_{r}∙{}^{\gamma }S$

This reduced model for blood clotting on a collagen/TF surface under flow uses 19 parameters, only 3 of which were adjusted to fit the experimental data:

7 kinetic coefficients (α_i_) based on measured kinetics and plasma zymogen levels (Table 1)
1 initial surface TF* level based on specified [TF]_o_ = 1 TF/μm^2^ and [FVIIa]/[FVII] = 0.01.
3 binding parameters: ^E^K_D_, ^γ^K_D_, k_f_
2 known stoichiometric coefficients: 1.6 E-sites/monomer, 0.3 γ’-sites/monomer
1 elution rate: k_elute_ = ln(2)/2s for free species of thrombin and FXIa
2 inhibition rates: k_i_ = ln(2)/60s for FXa, FIXa, FXIa, FIIa; k_i,TF_ = ln(2)/180s for TF*
3 effectiveness factors (η_4_, η_5_, η_6_) ≠ 1, adjusted to fit experimental data.

### Diffusion of thrombin from fibrin layer into a flow field

For simulations of thrombin equilibrated to fibrin (no thrombin generation) followed by desorption-controlled elution of thrombin, a full PDE simulation was solved for a 2D rectangular domain (1000 μm long x 60 μm high) representing a channel of the microfluidic device [13]. At a location 150 μm downstream of the entrance, a porous fibrin reaction zone (250-μm long x 15-μm high) was defined as the clot core, with fibrin concentration set by D-dimer ELISA experiment. A thrombin flux was imposed along the bottom plate to allow thrombin diffusion through the fibrin (in the presence of two binding sites (1.6 ^E^θ-sites per fibrin monomer; 0.3 ^γ^θ−sites per fibrin monomer) with binding kinetic parameters given Table 1. COMSOL was used to solve the convection-diffusion-reaction equation for thrombin transport in two steps. First, the Free and Porous Media Flow module was first solved with a Stationary study step to get the velocity field (*u*). Second, the mass transport was solved by the Transport of Diluted Species in Porous Media (thrombin) coupled with General Form PDE for weak and tight thrombin binding was solved with a time-varying time-step, with a relative tolerance of 0.0001.

The thrombin binding by fibrin was described by the following equations:

$$\frac{\partial C_{IIa}}{\partial t}=-\nabla ∙(-D\nabla C_{IIa})-u∙\nabla C_{IIa}-\frac{\partial^{E}S}{\partial t}-\frac{\partial^{\gamma }S}{\partial t}$$

$$\frac{\partial^{E}S}{\partial t}={}^{E}k_{f}∙C_{IIa}({}^{E}\theta_{total}-{}^{E}S)-{}^{E}k_{r}∙{}^{E}S$$

$$\frac{\partial^{\gamma }S}{\partial t}={}^{\gamma }k_{f}∙C_{IIa}({}^{\gamma }\theta_{total}-{}^{\gamma }S)-{}^{\gamma }k_{r}∙{}^{\gamma }S$$
